# 2,3,9,10,15,16-Hexaaza­tetra­cyclo­[6.6.2.0^4,16^.0^11,15^]hexa­decane dihydrate

**DOI:** 10.1107/S1600536812030735

**Published:** 2012-07-25

**Authors:** Fiona N.-F. How, Z. A. Rahima, Yee Seng Tan, S. Nadiah Abdul Halim, Seik Weng Ng

**Affiliations:** a Department of Biotechnology, Kulliyyah of Science, International Islamic University Malaysia, 25200 Kuantan, Pahang Darul Makmur, Malaysia; b Department of Chemistry, University of Malaya, 50603 Kuala Lumpur, Malaysia; c Chemistry Department, King Abdulaziz University, PO Box 80203 Jeddah, Saudi Arabia

## Abstract

The four six-membered fused rings in the title compound, C_10_H_20_N_6_·2H_2_O, adopt chair conformations; the H atoms of the four secondary N atoms occupy axial positions. Hydrogen bonds of the types N—H⋯N, N—H⋯O and O—H⋯N link the organic and water mol­ecules into a three-dimensional network.

## Related literature

For background to the reaction of glutaraldehyde and monosubstituted hydrazines, see: Katritzky & Fan (1990 ▶).
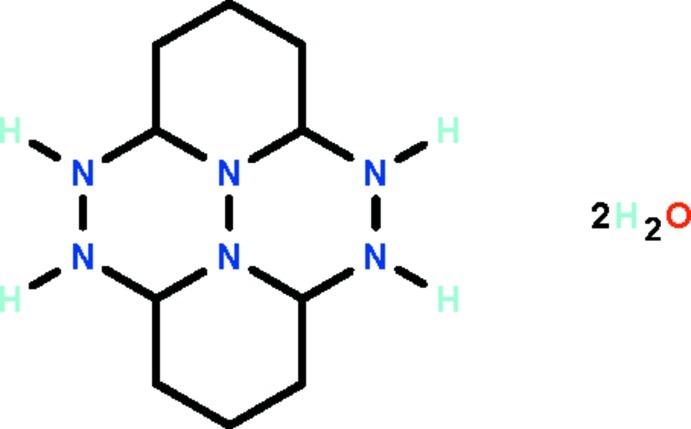


## Experimental

### 

#### Crystal data

C_10_H_20_N_6_·2H_2_O
*M* *_r_* = 260.35
Monoclinic, 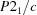

*a* = 9.5154 (10) Å
*b* = 16.0667 (17) Å
*c* = 9.1097 (10) Å
β = 114.916 (1)°
*V* = 1263.1 (2) Å^3^
*Z* = 4
Mo *K*α radiation
μ = 0.10 mm^−1^
*T* = 100 K
0.20 × 0.20 × 0.05 mm

#### Data collection

Bruker SMART APEX diffractometer
14226 measured reflections
2896 independent reflections
2138 reflections with *I* > 2σ(*I*)
*R*_int_ = 0.048

#### Refinement

*R*[*F* ^2^ > 2σ(*F* ^2^)] = 0.043
*wR*(*F* ^2^) = 0.116
*S* = 1.02
2896 reflections
195 parameters
H atoms treated by a mixture of independent and constrained refinement
Δρ_max_ = 0.31 e Å^−3^
Δρ_min_ = −0.23 e Å^−3^

### 

Data collection: *APEX2* (Bruker, 2009 ▶); cell refinement: *SAINT* (Bruker, 2009 ▶); data reduction: *SAINT*; program(s) used to solve structure: *SHELXS97* (Sheldrick, 2008 ▶); program(s) used to refine structure: *SHELXL97* (Sheldrick, 2008 ▶); molecular graphics: *X-SEED* (Barbour, 2001 ▶); software used to prepare material for publication: *publCIF* (Westrip, 2010 ▶).

## Supplementary Material

Crystal structure: contains datablock(s) global, I. DOI: 10.1107/S1600536812030735/bt5969sup1.cif

Structure factors: contains datablock(s) I. DOI: 10.1107/S1600536812030735/bt5969Isup2.hkl

Supplementary material file. DOI: 10.1107/S1600536812030735/bt5969Isup3.cml

Additional supplementary materials:  crystallographic information; 3D view; checkCIF report

## Figures and Tables

**Table 1:** Hydrogen-bond geometry (Å, °)

*D*—H⋯*A*	*D*—H	H⋯*A*	*D*⋯*A*	*D*—H⋯*A*
O1*W*—H11⋯N3^i^	0.93 (3)	1.99 (3)	2.922 (2)	174 (2)
O1*W*—H12⋯N5^ii^	0.92 (3)	2.05 (3)	2.960 (2)	178 (2)
O2*W*—H21⋯N2	0.88 (3)	2.05 (3)	2.925 (2)	172 (2)
O2*W*—H22⋯N4^ii^	0.86 (3)	2.02 (3)	2.863 (2)	167 (2)
N1—H1⋯O1*W*	0.88 (2)	2.08 (2)	2.964 (2)	175 (2)
N2—H2⋯O2*W* ^iii^	0.91 (2)	2.18 (2)	3.071 (2)	166 (2)
N4—H4⋯N5^iv^	0.87 (2)	2.57 (2)	3.354 (2)	150.1 (15)
N5—H5⋯N1^iii^	0.96 (2)	2.25 (2)	3.163 (2)	159 (2)

Symmetry codes: (i) 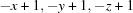; (ii) 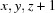; (iii) 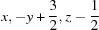; (iv) 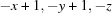.

**Fig. 1.**
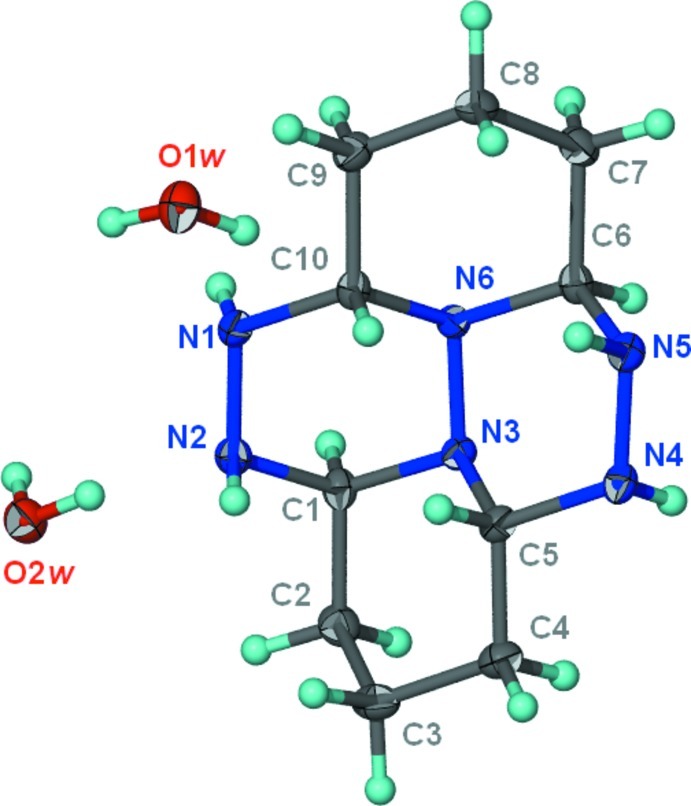
Anisotropic displacement ellipsoid plot (Barbour, 2001) of C10H20N6.2H2O at the 70% probability level; hydrogen atoms are drawn as spheres of arbitrary radius.

## supplementary crystallographic information

### Comment

Glutaraldehyde, CH═O(CH_2_)_3_CH═O, condenses with mono-substituted hydrazines to yield *N*-substituted piperidines; for example, it reacts with phenylhydrazine to yield *N*-phenylpiperidin-1-amine (Katritzky & Fan, 1990). The direct reaction of the di-aldehyde with hydrazine itself does not lead to the formation of a polymeric Schiff-base product; the compound is, in fact, 2,3,9,10,15,16-hexaazatetracyclo[6,2,0^4,16^.0^11,15^]hexadecane, which crystallizes as a dihydrate (Scheme I). The four six-membered fused rings adopt chair conformations (Fig. 1). Hydrogen bonds of the type N–H···N, N–H···O and O–H···N link the organic and water molecules into a three dimensional network (Table 1).

There is no precedent for the fused-ring system in the crystallographic literature.

### Experimental

Hydrazine hydrate (0.08 mol, 2.7 ml) was added to glutaraldehyde (0.08 g, 8.1 ml) to give a white solid. and stirred at room temperature to yield a white solid. This was recrystallized from ethanol; several drops of DMSO was added to aid crystallization.

### Refinement

Carbon-bound H-atoms were placed in calculated positions (C—H 0.99 to 1.00 Å) and were included in the refinement in the riding model approximation, with *U*(H) set to 1.2 *U*(C).

The amino and water H-atoms were located in a difference Fourier map, and were freely refined.

### Crystal data

**Table:**

C_10_H_20_N_6_·2H_2_O	*F*(000) = 568
*M**_r_* = 260.35	*D*_x_ = 1.369 Mg m^−^^3^
Monoclinic, *P*2_1_/*c*	Mo *K*α radiation, λ = 0.71073 Å
Hall symbol: -P 2ybc	Cell parameters from 2285 reflections
*a* = 9.5154 (10) Å	θ = 2.3–30.0°
*b* = 16.0667 (17) Å	µ = 0.10 mm^−^^1^
*c* = 9.1097 (10) Å	*T* = 100 K
β = 114.916 (1)°	Prism, colorless
*V* = 1263.1 (2) Å^3^	0.20 × 0.20 × 0.05 mm
*Z* = 4	

### Data collection

**Table:**

Bruker SMART APEX diffractometer	2138 reflections with *I* > 2σ(*I*)
Radiation source: fine-focus sealed tube	*R*_int_ = 0.048
Graphite monochromator	θ_max_ = 27.5°, θ_min_ = 2.5°
ω scans	*h* = −12→12
14226 measured reflections	*k* = −20→20
2896 independent reflections	*l* = −11→11

### Refinement

**Table:**

Refinement on *F*^2^	Primary atom site location: structure-invariant direct methods
Least-squares matrix: full	Secondary atom site location: difference Fourier map
*R*[*F*^2^ > 2σ(*F*^2^)] = 0.043	Hydrogen site location: inferred from neighbouring sites
*wR*(*F*^2^) = 0.116	H atoms treated by a mixture of independent and constrained refinement
*S* = 1.02	*w* = 1/[σ^2^(*F*_o_^2^) + (0.0493*P*)^2^ + 0.6276*P*] where *P* = (*F*_o_^2^ + 2*F*_c_^2^)/3
2896 reflections	(Δ/σ)_max_ = 0.001
195 parameters	Δρ_max_ = 0.31 e Å^−^^3^
0 restraints	Δρ_min_ = −0.23 e Å^−^^3^

### Fractional atomic coordinates and isotropic or equivalent isotropic displacement parameters (Å^2^)

**Table:**

	*x*	*y*	*z*	*U*_iso_*/*U*_eq_	
O1W	0.34037 (14)	0.59785 (8)	0.67888 (15)	0.0195 (3)	
O2W	0.75367 (14)	0.67859 (7)	0.93500 (16)	0.0182 (3)	
N1	0.47258 (15)	0.70228 (8)	0.49990 (16)	0.0129 (3)	
N2	0.63553 (16)	0.68083 (8)	0.58207 (16)	0.0134 (3)	
N3	0.60274 (15)	0.57645 (8)	0.36983 (15)	0.0115 (3)	
N4	0.61057 (16)	0.59177 (9)	0.11065 (16)	0.0138 (3)	
N5	0.44386 (15)	0.60328 (8)	0.03409 (16)	0.0136 (3)	
N6	0.43683 (15)	0.59445 (8)	0.30232 (15)	0.0111 (3)	
C1	0.66679 (19)	0.59553 (9)	0.54388 (18)	0.0132 (3)	
H1A	0.6175	0.5564	0.5937	0.016*	
C2	0.84013 (19)	0.57828 (10)	0.6218 (2)	0.0170 (4)	
H2A	0.8576	0.5176	0.6203	0.020*	
H2B	0.8837	0.5965	0.7362	0.020*	
C3	0.92503 (19)	0.62308 (10)	0.5346 (2)	0.0177 (4)	
H3A	0.9182	0.6840	0.5462	0.021*	
H3B	1.0358	0.6071	0.5839	0.021*	
C4	0.85215 (18)	0.59957 (10)	0.3555 (2)	0.0166 (3)	
H4A	0.9039	0.6307	0.2984	0.020*	
H4B	0.8673	0.5394	0.3439	0.020*	
C5	0.68021 (18)	0.61943 (9)	0.27960 (18)	0.0128 (3)	
H5A	0.6657	0.6809	0.2837	0.015*	
C6	0.36997 (18)	0.56712 (10)	0.13338 (19)	0.0130 (3)	
H6	0.3851	0.5055	0.1344	0.016*	
C7	0.19610 (19)	0.58261 (10)	0.0546 (2)	0.0168 (4)	
H7A	0.1451	0.5442	0.1021	0.020*	
H7B	0.1556	0.5703	−0.0626	0.020*	
C8	0.15525 (19)	0.67222 (10)	0.0773 (2)	0.0170 (4)	
H8A	0.1920	0.7104	0.0157	0.020*	
H8B	0.0414	0.6781	0.0353	0.020*	
C9	0.23099 (19)	0.69524 (10)	0.25679 (19)	0.0156 (3)	
H9A	0.2083	0.7542	0.2700	0.019*	
H9B	0.1867	0.6604	0.3165	0.019*	
C10	0.40528 (18)	0.68252 (9)	0.32748 (18)	0.0125 (3)	
H10A	0.4508	0.7199	0.2711	0.015*	
H1	0.427 (2)	0.6716 (12)	0.548 (2)	0.018 (5)*	
H2	0.687 (2)	0.7182 (13)	0.547 (2)	0.023 (5)*	
H4	0.629 (2)	0.5389 (14)	0.108 (2)	0.027 (5)*	
H5	0.427 (2)	0.6621 (13)	0.024 (2)	0.024 (5)*	
H11	0.364 (3)	0.5434 (17)	0.661 (3)	0.053 (7)*	
H12	0.371 (3)	0.5987 (15)	0.789 (3)	0.051 (7)*	
H21	0.711 (3)	0.6761 (14)	0.829 (3)	0.039 (7)*	
H22	0.704 (3)	0.6475 (15)	0.974 (3)	0.045 (7)*	

### Atomic displacement parameters (Å^2^)

**Table:**

	*U*^11^	*U*^22^	*U*^33^	*U*^12^	*U*^13^	*U*^23^
O1W	0.0256 (7)	0.0162 (6)	0.0178 (6)	0.0021 (5)	0.0100 (5)	0.0008 (5)
O2W	0.0192 (7)	0.0204 (6)	0.0158 (6)	−0.0034 (5)	0.0083 (5)	−0.0005 (5)
N1	0.0130 (7)	0.0144 (7)	0.0120 (6)	0.0006 (5)	0.0060 (6)	−0.0009 (5)
N2	0.0135 (7)	0.0115 (6)	0.0147 (7)	−0.0002 (5)	0.0054 (6)	−0.0009 (5)
N3	0.0114 (7)	0.0122 (6)	0.0118 (6)	0.0001 (5)	0.0057 (5)	0.0003 (5)
N4	0.0162 (7)	0.0121 (7)	0.0137 (7)	0.0023 (5)	0.0070 (6)	−0.0006 (5)
N5	0.0149 (7)	0.0134 (7)	0.0128 (7)	0.0014 (5)	0.0060 (6)	0.0006 (5)
N6	0.0106 (6)	0.0101 (6)	0.0117 (6)	0.0007 (5)	0.0040 (5)	−0.0005 (5)
C1	0.0166 (8)	0.0111 (7)	0.0119 (7)	−0.0001 (6)	0.0060 (6)	−0.0002 (6)
C2	0.0162 (8)	0.0156 (8)	0.0163 (8)	0.0016 (6)	0.0041 (7)	−0.0002 (6)
C3	0.0121 (8)	0.0171 (8)	0.0220 (9)	0.0017 (6)	0.0055 (7)	0.0011 (6)
C4	0.0142 (8)	0.0171 (8)	0.0199 (8)	0.0012 (6)	0.0084 (7)	0.0005 (6)
C5	0.0137 (8)	0.0121 (7)	0.0145 (8)	0.0007 (6)	0.0076 (6)	0.0004 (6)
C6	0.0159 (8)	0.0112 (7)	0.0122 (7)	−0.0010 (6)	0.0061 (6)	−0.0006 (6)
C7	0.0147 (8)	0.0191 (8)	0.0147 (8)	−0.0030 (6)	0.0044 (7)	−0.0020 (6)
C8	0.0120 (8)	0.0194 (8)	0.0177 (8)	0.0020 (6)	0.0043 (7)	−0.0005 (6)
C9	0.0153 (8)	0.0161 (8)	0.0160 (8)	0.0023 (6)	0.0073 (7)	0.0003 (6)
C10	0.0149 (8)	0.0108 (7)	0.0127 (8)	0.0005 (6)	0.0066 (6)	−0.0003 (6)

### Geometric parameters (Å, º)

**Table:**

O1W—H11	0.93 (3)	C2—H2A	0.9900
O1W—H12	0.92 (3)	C2—H2B	0.9900
O2W—H21	0.88 (3)	C3—C4	1.528 (2)
O2W—H22	0.86 (3)	C3—H3A	0.9900
N1—N2	1.4509 (19)	C3—H3B	0.9900
N1—C10	1.460 (2)	C4—C5	1.518 (2)
N1—H1	0.88 (2)	C4—H4A	0.9900
N2—C1	1.4748 (19)	C4—H4B	0.9900
N2—H2	0.91 (2)	C5—H5A	1.0000
N3—N6	1.4615 (17)	C6—C7	1.521 (2)
N3—C1	1.4715 (19)	C6—H6	1.0000
N3—C5	1.4853 (19)	C7—C8	1.528 (2)
N4—N5	1.4508 (19)	C7—H7A	0.9900
N4—C5	1.465 (2)	C7—H7B	0.9900
N4—H4	0.87 (2)	C8—C9	1.528 (2)
N5—C6	1.479 (2)	C8—H8A	0.9900
N5—H5	0.96 (2)	C8—H8B	0.9900
N6—C6	1.4633 (19)	C9—C10	1.519 (2)
N6—C10	1.4843 (19)	C9—H9A	0.9900
C1—C2	1.521 (2)	C9—H9B	0.9900
C1—H1A	1.0000	C10—H10A	1.0000
C2—C3	1.530 (2)		
			
H11—O1W—H12	103 (2)	C3—C4—H4A	109.5
H21—O2W—H22	110 (2)	C5—C4—H4B	109.5
N2—N1—C10	113.12 (12)	C3—C4—H4B	109.5
N2—N1—H1	104.6 (12)	H4A—C4—H4B	108.1
C10—N1—H1	109.1 (13)	N4—C5—N3	109.28 (12)
N1—N2—C1	112.14 (12)	N4—C5—C4	109.79 (13)
N1—N2—H2	106.1 (12)	N3—C5—C4	109.95 (13)
C1—N2—H2	109.9 (13)	N4—C5—H5A	109.3
N6—N3—C1	107.21 (11)	N3—C5—H5A	109.3
N6—N3—C5	112.03 (11)	C4—C5—H5A	109.3
C1—N3—C5	115.01 (12)	N6—C6—N5	114.67 (12)
N5—N4—C5	112.67 (12)	N6—C6—C7	110.60 (13)
N5—N4—H4	107.5 (13)	N5—C6—C7	110.49 (13)
C5—N4—H4	108.6 (13)	N6—C6—H6	106.9
N4—N5—C6	111.18 (12)	N5—C6—H6	106.9
N4—N5—H5	106.0 (12)	C7—C6—H6	106.9
C6—N5—H5	110.3 (12)	C6—C7—C8	112.24 (13)
N3—N6—C6	107.29 (11)	C6—C7—H7A	109.2
N3—N6—C10	112.12 (11)	C8—C7—H7A	109.2
C6—N6—C10	114.95 (12)	C6—C7—H7B	109.2
N3—C1—N2	114.49 (12)	C8—C7—H7B	109.2
N3—C1—C2	109.88 (13)	H7A—C7—H7B	107.9
N2—C1—C2	110.24 (13)	C9—C8—C7	109.92 (13)
N3—C1—H1A	107.3	C9—C8—H8A	109.7
N2—C1—H1A	107.3	C7—C8—H8A	109.7
C2—C1—H1A	107.3	C9—C8—H8B	109.7
C1—C2—C3	112.22 (13)	C7—C8—H8B	109.7
C1—C2—H2A	109.2	H8A—C8—H8B	108.2
C3—C2—H2A	109.2	C10—C9—C8	111.07 (13)
C1—C2—H2B	109.2	C10—C9—H9A	109.4
C3—C2—H2B	109.2	C8—C9—H9A	109.4
H2A—C2—H2B	107.9	C10—C9—H9B	109.4
C4—C3—C2	109.67 (14)	C8—C9—H9B	109.4
C4—C3—H3A	109.7	H9A—C9—H9B	108.0
C2—C3—H3A	109.7	N1—C10—N6	110.36 (12)
C4—C3—H3B	109.7	N1—C10—C9	109.43 (13)
C2—C3—H3B	109.7	N6—C10—C9	108.58 (12)
H3A—C3—H3B	108.2	N1—C10—H10A	109.5
C5—C4—C3	110.60 (13)	N6—C10—H10A	109.5
C5—C4—H4A	109.5	C9—C10—H10A	109.5
			
C10—N1—N2—C1	−48.61 (17)	C1—N3—C5—C4	56.60 (16)
C5—N4—N5—C6	−50.99 (16)	C3—C4—C5—N4	−176.52 (13)
C1—N3—N6—C6	−174.01 (11)	C3—C4—C5—N3	−56.27 (17)
C5—N3—N6—C6	58.93 (14)	N3—N6—C6—N5	−55.08 (16)
C1—N3—N6—C10	58.87 (15)	C10—N6—C6—N5	70.36 (17)
C5—N3—N6—C10	−68.19 (15)	N3—N6—C6—C7	179.18 (12)
N6—N3—C1—N2	−55.16 (16)	C10—N6—C6—C7	−55.38 (17)
C5—N3—C1—N2	70.13 (17)	N4—N5—C6—N6	52.05 (17)
N6—N3—C1—C2	−179.84 (12)	N4—N5—C6—C7	177.85 (12)
C5—N3—C1—C2	−54.55 (16)	N6—C6—C7—C8	51.81 (18)
N1—N2—C1—N3	51.03 (17)	N5—C6—C7—C8	−76.24 (17)
N1—N2—C1—C2	175.52 (12)	C6—C7—C8—C9	−53.06 (18)
N3—C1—C2—C3	53.32 (17)	C7—C8—C9—C10	56.33 (18)
N2—C1—C2—C3	−73.78 (17)	N2—N1—C10—N6	52.49 (17)
C1—C2—C3—C4	−55.36 (17)	N2—N1—C10—C9	171.91 (12)
C2—C3—C4—C5	56.48 (17)	N3—N6—C10—N1	−59.00 (16)
N5—N4—C5—N3	55.05 (16)	C6—N6—C10—N1	178.11 (12)
N5—N4—C5—C4	175.72 (12)	N3—N6—C10—C9	−178.95 (11)
N6—N3—C5—N4	−60.10 (15)	C6—N6—C10—C9	58.17 (16)
C1—N3—C5—N4	177.16 (12)	C8—C9—C10—N1	−178.00 (13)
N6—N3—C5—C4	179.34 (12)	C8—C9—C10—N6	−57.48 (17)

### Hydrogen-bond geometry (Å, º)

**Table:**

*D*—H···*A*	*D*—H	H···*A*	*D*···*A*	*D*—H···*A*
O1*W*—H11···N3^i^	0.93 (3)	1.99 (3)	2.922 (2)	174 (2)
O1*W*—H12···N5^ii^	0.92 (3)	2.05 (3)	2.960 (2)	178 (2)
O2*W*—H21···N2	0.88 (3)	2.05 (3)	2.925 (2)	172 (2)
O2*W*—H22···N4^ii^	0.86 (3)	2.02 (3)	2.863 (2)	167 (2)
N1—H1···O1*W*	0.88 (2)	2.08 (2)	2.964 (2)	175 (2)
N2—H2···O2*W*^iii^	0.91 (2)	2.18 (2)	3.071 (2)	166 (2)
N4—H4···N5^iv^	0.87 (2)	2.57 (2)	3.354 (2)	150.1 (15)
N5—H5···N1^iii^	0.96 (2)	2.25 (2)	3.163 (2)	159 (2)

Symmetry codes: (i) −*x*+1, −*y*+1, −*z*+1; (ii) *x*, *y*, *z*+1; (iii) *x*, −*y*+3/2, *z*−1/2; (iv) −*x*+1, −*y*+1, −*z*.
